# Derivation of Indices of Cognitive Change Among Hispanic Adults and Elders

**DOI:** 10.1001/jamanetworkopen.2024.31180

**Published:** 2024-09-03

**Authors:** Kevin Duff, Justin B. Miller, Kim Cobos, Jessica Rodrigues, Sid E. O’Bryant

**Affiliations:** 1 Layton Aging & Alzheimer Disease Center, Oregon Health and Science University, Portland; 2 Cleveland Clinic Lou Ruvo Center for Brain Health, Las Vegas, Nevada; 3 Institute for Translational Research, University of North Texas Health Science Center, Fort Worth

## Abstract

**Importance:**

Determining the influence of race and ethnicity on change in cognitive test performance has significant implications for clinical practice and research in populations at risk for Alzheimer disease.

**Objective:**

To evaluate the significance of race and ethnicity in predicting longitudinal cognitive test performance and to develop models to support evidence-based practice.

**Design, Setting, and Participants:**

This prognostic study included baseline and 24-month follow-up data that were obtained from the Health and Aging Brain Study—Health Disparities (HABS-HD) study, an ongoing longitudinal observational study of aging and dementia in a multiracial, multiethnic cohort. Participants included community-dwelling adults and elders living in the Dallas and Fort Worth metropolitan area who were Hispanic and non-Hispanic adults older than the age of 50 years and were cognitively unimpaired.

**Exposure:**

The primary exposure of interest was time, measured in months.

**Main Outcomes and Measures:**

Demographic variables included age, sex, education, and race and ethnicity. Cognitive domains included attention and working memory, processing speed, language, memory, and executive functioning. Linear regression models predicted follow-up performance from baseline performance and demographic variables for 13 commonly used neuropsychological tests. Follow-up testing was the primary outcome for all domains. Raw scores from 13 standardized tests were used for analyses.

**Results:**

This study included 799 adults who were cognitively unimpaired (352 Hispanic individuals [44.1%]; 447 non-Hispanic individuals [55.9%]; 524 female [65.6%]; mean [SD] age, 65.4 [8.1] years). In the regression models, all 13 follow-up scores were significantly predicted from their respective baseline scores and demographic variables. Baseline performance and education were the most consistent predictors of follow-up scores, contributing to all 13 models. Age was significantly associated with follow-up in 11 models, and sex was significant in 5 models. Race and ethnicity contributed to 10 of 13 models, with Hispanic participants predicted to have poorer follow-up scores than their non-Hispanic White counterparts on each test.

**Conclusions and Relevance:**

In this longitudinal study of cognitive change in Hispanic and non-Hispanic older adults who were cognitively unimpaired, standardized regression-based models were influenced by multiple demographic variables, including race and ethnicity. These findings highlight the importance of including race and ethnicity in such cognitive change models. This ability to accurately predict cognitive change is expected to become increasingly important as clinical practice and clinical trials need to become more diverse and culturally appropriate in this burgeoning global medical and societal crisis.

## Key Points

**Question:**

Are race and ethnicity important predictors in determining the significance of cognitive change over time among older adults?

**Findings:**

In this prognostic study of 799 cognitively unimpaired older adults who were seen at 2 time points 24 months apart, race and ethnicity were significantly associated with change over time for 10 of 13 commonly used cognitive tests.

**Meaning:**

These findings suggest that demographic factors, including race and ethnicity, provided prognostic value in determining how an individual’s cognitive test scores can change over time and need to be considered in settings designed to assess for change in cognitive test performance.

## Teaser

This prognostic study examines the contribution of ethnicity in predicting longitudinal cognitive test performance among Hispanic and non-Hispanic adults aged 50 years and older.

## Introduction

Neuropsychological evaluations may be repeated in clinical and research settings to track decline due to a neurodegenerative condition or gauge efficacy on primary outcomes in clinical trials. Whereas normative data are abundant for baseline testing, fewer resources are available for determining statistically and clinically meaningful change in test performance. In Alzheimer disease and related dementia (ADRD), identifying meaningful change is important for diagnostic decisions and treatment recommendations. Given new era disease modifying therapies for ADRD,^1,2^ it is especially important to have tools to monitor change.

Multiple methods to evaluate change over time have been developed,^3^ and each have strengths and weaknesses. The simple difference method calculates the difference between scores at 2 time points. Although it is easy to calculate, it fails to account for confounding test operating characteristics like test-retest reliability, standard error of measurement, and practice effects. The Reliable Change Index (RCI)^4^ accounts for the reliability of scores but fails to account for regression to the mean and practice effects. An RCI that accounts practice effects was developed^5^; however, none of these methods account for demographic factors (eg, age, education) that can influence change over time.^6^

Standardized regression-based (SRB) methods potentially address most of these issues.^7^ Using performance at 1 time to predict performance at a subsequent time point, reliability and regression to the mean are controlled for. Additional predictors (eg, age, education, retest interval) can be incorporated to account for additional variance in change over time. The predicted follow-up scores are compared with observed follow-up scores and standardized to gauge the relative frequency of the difference. Thresholds for interpretation and identifying meaningful change can be applied. Typically developed in normal individuals, SRBs have been published for mild cognitive impairment (MCI) and dementia.^8,9^ Despite their benefits, development of SRB formulas requires data from large longitudinal cohorts.

A major gap in current knowledge is how change over time varies as a function of race and ethnicity. Race typically refers to the categorization of people based on perceived shared physical traits, whereas ethnicity categorizes people based on shared culture related to common ancestry.^10^ These categorizations are important considerations when interpreting cognitive diagnoses and test performances in longitudinal studies. For example, Latinx individuals have an increased risk of conversion to dementia compared with non-Latinx White participants.^11^ Steeper memory declines over time have been noted in older Black women compared with non-Hispanic White older women.^12^ Older African American individuals experienced faster decline in verbal memory compared with White individuals, with race accounting for 7% of the yearly rate of change.^13^ These studies highlight the limited literature on race and ethnicity influencing change over time, despite ample evidence that Black and Hispanic older adults are at greater risk for ADRD.^14^ Although it is important to appreciate that cognitive disparities arise from complex sociocultural and environmental factors (eg, test bias, quality of education, neighborhood disadvantage, discrimination),^15^ it is critically important to develop tools to determine the significance of change over time in different racial and ethnic groups.

Using data from the Health and Aging Brain Study–Health Disparities (HABS-HD; formally the Health and Aging Brain Study Among Latino Elders) project, we aim to fill this gap by developing SRB methods for identifying clinically meaningful change in Hispanic older adults. It was hypothesized that race and ethnicity would significantly predict longitudinal change on cognitive scores. Achieving these goals can improve care by bolstering the evidence-base used in clinical decision-making, as well as broadening research opportunities for this growing segment of the population.

## Methods

### Participants

Data were obtained from the HABS-HD project, a longitudinal, community-based project examining health and aging disparities in 3 groups of adults and older adults: Black and African American, Hispanic, or non-Hispanic White (however, the Black and African American group was not used because longitudinal data are not yet available). The present analyses used Release 4 of the dataset (obtained on May 3, 2023). Inclusion criteria for HABS-HD included (1) self-reported race and ethnicity of Black and African American, Hispanic, or non-Hispanic White; (2) willingness to provide blood samples; (3) age 30 years and older; (4) fluent in English or Spanish; (5) willing and capable of undergoing magnetic resonance imaging (MRI) scan of the head; and (6) availability of a reliable informant. Exclusion criteria included (1) type 1 diabetes; (2) active uncontrolled inflammatory condition; (3) current or recent cancer (besides skin cancer); (4) current severe mental illness that could impact cognition, except depression; (5) recent traumatic brain injury with loss of consciousness; (6) current or recent alcohol or substance abuse; and (7) active severe medical condition that could impact cognition. Individuals with baseline and at least 1 follow-up visit were included in these analyses.

### Procedures

This diagnostic/prognostic study follows the Standards for Reporting of Diagnostic Accuracy (STARD) reporting guideline.^16^ The HABS-HD study has been approved by the Institutional Review Board at the University of North Texas, Health Science Center prior to study commencement, and each participant (or his/her legal representative) provided informed consent prior to data collection that stipulates sharing of deidentified data. HABS-HD includes an interview, brief functional examination, blood draw, neuropsychological testing, and MRI of the brain. Study procedures were conducted in Spanish or English, based on participant preference. However, language proficiency was not formally assessed, all individuals were asked for the language that they wished to be tested in, and all testing was completed in the chosen language. Participants were examined at baseline (T1) and follow-up visits (T2), which were approximately 24 months apart.

The neuropsychological testing battery included: Mini Mental Status Exam (MMSE, total score), Spanish-English Verbal Learning Test (SEVLT: total recall of 15 words learned across 5 trials and 30-minute delayed recall), Wechsler Memory Scale-III (WMS-III) Logical Memory (immediate recall and 30-minute delayed recall) and Digit Span (forward, backward, and total), Trail Making Test (TMT) Parts A and B (time to completion), phonemic fluency (F, A, and S for 60 seconds trials), semantic fluency (animals in 60 seconds), and Digit Symbol Substitution (correct in 90 seconds). Raw scores were used in analyses and higher scores indicated better cognitive functioning, with the exception of TMT. The Clinical Dementia Rating Scale (CDR)^17^ was completed with an informant to evaluate for functional declines. For the CDR, the Sum of Box scores was used, with higher scores indicating greater impairment.

Cognitive diagnoses were assigned using a decision tree and verified via consensus review. Individuals classified as cognitively unimpaired (CU) did not report any cognitive complaints, had a CDR of 0, and had cognitive scores broadly within normal limits (ie, performance better than MCI). To be classified as MCI, individuals reported cognitive complaints (participant or informant), had a CDR of 0.5 to 2.0, and had at least 1 cognitive score falling 1.5 or more SDs below normative ranges. Normative data for the cognitive scores^18^ corrected for age (65 years and younger and 66 years and older), education (0 to 7 years, 8 to 12 years, and 13 years or more), and primary language (English or Spanish). This same normative data were used for both Hispanic and non-Hispanic White participants. For this study, only individuals classified as CU at both T1 and T2 were included in these analyses.

### Statistical Analysis

In primary analyses, multivariable linear regression was used to develop SRBs for the 13 cognitive test scores. In each model, performance at T2 score was the dependent variable, and the corresponding performance at T1, age, education, sex, and race and ethnicity (non-Hispanic White or Hispanic) were included as predictors. Predictors were entered simultaneously to generate more stable models. Models were bootstrapped on 1000 samples. Within each model, the bootstrapped coefficients identified the influence of the predictors on the T2 scores. Once developed, these SRB models were applied to the intact sample and *z *scores were generated for each individual for each of the 13 cognitive scores. Positive *z *scores reflected better performance at T2 than predicted based on the model, and negative *z *scores reflected worse than predicted performance at T2. Group differences between the non-Hispanic White and Hispanic groups on those 13 SRB-derived *z *scores were then explored using independent sample *t* tests. Additionally, the individual *z *scores were categorized as decline (*z* ≤ −1.645), stable (−1.644 > *z* < 1.644), or improve (*z* ≥ 1.645) for each participant and for each cognitive score. These categorized scores were then compared via χ^2^ analyses for non-Hispanic White and Hispanic individuals to determine the proportion of individuals in each category as a function of race and ethnicity. In secondary analyses, regression models were repeated using only data from the non-Hispanic White group to examine the effects of not including racial and ethnic status in the SRB models. *t* Tests and χ^2^ tests compared the non-Hispanic White and Hispanic participants on these nonracially or ethnically corrected SRB scores. Data were analyzed from May 3, 2023, to July 1, 2024. Statistical significance was set at *P* < .001, tests were 2-sided, and analyses were performed using SPSS Software version 29.0 (IBM).

## Results

Of the 799 individuals classified as CU at both T1 and T2, 447 (55.9%) were non-Hispanic White participants and 352 (44.1%) were Hispanic participants, and their mean (SD) age was 65.4 (8.1) years old, mean (SD) education was 13.4 (4.3) years, and 524 were female (65.6%) (Table 1). Non-Hispanic White participants were significantly older (t_797_ = 10.23; *P* < .001), had more years of education (t_797_ = 23.03; *P* < .001), and had a higher percentage of males (*X*^2^_1,n=799_ = 17.829, *P* < .001) than their Hispanic counterparts. For all cognitive scores at T1, the non-Hispanic White participants performed significantly better than the Hispanic participants (Table 2). Non-Hispanic White participants endorsed fewer symptoms of depression (mean [SD], 4.0 [4.6]; range, 0-29) at T1 than their Hispanic peers (mean [SD], 5.8 [6.1]; range, 0-29) (*P* < .001).

**Table 1.** Baseline Demographic Information by Group

Characteristics	Participants, No. (%)	
	Hispanic (n = 352)	Non-Hispanic White (n = 447)
Age, mean (SD), y^a^	62.3 (7.3)	67.9 (7.9)
Education, mean (SD), y^a^	10.3 (4.3)	15.8 (2.4)
Sex		
Male^a^	93 (26.4)	182 (40.7)
Female^a^	259 (73.6)	265 (59.3)

^a^ *P* < .001.

**Table 2.** Test Performance Data by Group at Time 1 and Time 2

Group	Hispanic				Non-Hispanic White			
	Mean (SD)		*P* value	*d*	Mean (SD)		*P* value	*d*
	Time 1	Time 2			Time 1	Time 2		
MMSE total	27.43 (2.20)	27.50 (2.27)	.49	−0.04	29.26 (0.96)	29.09 (1.17)	.004	0.14
SEVLT learning	32.32 (7.22)	33.27 (7.96)	.01	−0.15	36.24 (7.24)	36.57 (7.73)	.27	−0.53
SEVLT delay	8.36 (2.64)	8.71 (2.79)	.01	−0.14	9.67 (2.62)	9.81 (2.73)	.22	−0.06
Logical memory immediate	35.39 (9.08)	36.86 (8.48)	.001	−0.22	44.00 (8.90)	44.76 (8.50)	.03	−0.10
Logical memory delay	22.14 (6.91)	23.72 (6.88)	.001	−0.30	27.62 (6.91)	28.49 (6.58)	.001	−0.15
Digit span-forward	7.52 (2.09)	7.53 (1.96)	.88	−0.01	9.94 (2.01)	10.03 (2.12)	.26	−0.05
Digit span-backward	5.09 (1.85)	5.09 (1.76)	>.99	0	6.89 (1.93)	6.87 (1.99)	.83	0.01
Digit span-total	12.62 (3.53)	12.58 (3.32)	.73	0.02	16.85 (3.36)	16.80 (3.78)	.74	0.02
Trails A	40.68 (18.04)	41.24 (17.53)	.46	−0.04	31.62 (9.82)	31.11 (10.02)	.19	0.06
Trails B	126.79 (78.61)	125.56 (77.40)	.68	0.02	73.81 (29.39)	76.19 (34.14)	.09	−0.08
FAS	30.82 (10.16)	31.92 (10.59)	.001	−0.18	39.74 (10.48)	41.03 (10.72)	.001	−0.18
Animals	17.54 (4.52)	18.02 (4.50)	.01	−0.13	20.70 (5.98)	20.04 (4.57)	.02	0.12
Digit symbol subs.	39.65 (11.89)	39.54 (12.67)	.73	0.02	49.36 (9.60)	49.03 (9.92)	.24	0.07

Abbreviations: FAS, phonemic fluency; MMSE, Mini Mental Status Exam; SEVLT, Spanish English Verbal Learning Test.

### Primary Analyses

Model summary statistics are presented in Table 3. In the bootstrapped, multivariable linear regression models, all 13 T2 scores were significantly predicted from their respective T1 scores and demographic variables. When examining the individual predictors, T1 scores significantly predicted T2 scores in all 13 models (eg, MMSE total, unstandardized β, 0.48 [95% CI, 0.40 to 0.56]; *P* < .001). Because all bootstrapped coefficients were positive, higher T1 scores predicted higher T2 scores. Education also significantly contributed to all of the models (eg, SEVLT learning, unstandardized β, 0.26 [95% CI, 0.13 to 0.38];* P* < .001), with more education predicting better T2 scores. Age significantly contributed to 11 of the 13 models (eg, MMSE total, unstandardized β, −0.03 [95% CI, −0.04 to −0.02]; *P* < .001), with higher age predicting poorer T2 scores. Sex only significantly contributed to 4 of the 11 (eg, Logical Memory delay, unstandardized β, 1.15 [95% CI, 0.40 to 1.85]; *P* = .005, with females having better predicted T2 scores than males. Finally, race and ethnicity significantly contributed to 10 of 13 models (Trails A, unstandardized β, 3.26 [95% CI, 1.60 to 4.95]; *P* < .001), with the Hispanic individuals being predicted to have poorer T2 scores than non-Hispanic White individuals.

**Table 3.** Regression Model Summaries Predicting Time 2 Performance

Model	*R* ^2^	Constant	Predictors									
			Time 1		Age		Education		Sex		Ethnicity	
			Unstandardized β (95% CI)	Standardized β	Unstandardized β (95% CI)	Standardized β	Unstandardized β (95% CI)	Standardized β	Unstandardized β (95% CI)	Standardized β	Unstandardized β (95% CI)	Standardized β
MMSE Total	0.50	15.39 (12.68 to 17.95)	0.48 (0.40 to 0.56)	0.47	−0.03 (−0.04 to −0.02)^a^	−0.13	0.12 (0.08 to 0.15)^a^	0.26	0.05 (−0.14 to 0.23)	0.01	−0.25 (−0.48 to −0.02)^a^	−0.06
SEVLT Learning	0.51	23.76 (17.83 to 29.37)	0.58 (0.52 to 0.65)	0.54	−0.19 (−0.24 to −0.13)^a^	−0.19	0.26 (0.13 to 0.38)^a^	0.14	2.22 (1.33 to 3.10)^a^	0.13	−0.94 (−2.06 to 0.29)	−0.06
SEVLT Delay	0.43	8.10 (6.13 to 10.00)	0.51 (0.45 to 0.57)	0.49	−0.07 (−0.08 to −0.05)^a^	−0.19	0.08 (0.03 to 0.13)^a^	0.12	0.79 (0.42 to 1.11)^a^	0.13	−0.49 (−0.86 to −0.11)^a^	−0.09
Logical Memory Immediate	0.56	21.65 (14.97 to 27.76)	0.57 (0.52 to 0.62)	0.61	−0.09 (−0.15 to −0.03)^a^	−0.08	0.34(0.19 to 0.48)^a^	0.16	0.64 (−0.27 to 1.58)	0.03	−1.70 (−2.91 to −0.51)^a^	−0.09
Logical Memory Delay	0.54	15.11 (10.68 to 19.70)	0.59 (0.54 to 0.65)	0.62	−0.10 (−0.14 to −0.05)^a^	−0.11	0.24 (0.14 to 0.35)^a^	0.14	1.15 (0.40 to 1.85)^a^	0.08	−0.93 (−1.84 to −0.02)^a^	−0.07
Digit Span−Forward	0.63	3.87 (2.49 to 5.21)	0.64 (0.59 to 0.70)	0.64	−0.01 (−0.02 to 0.01)^a^	−0.04	0.08 (0.05 to 0.11)^a^	0.14	−0.17 (−0.41 to 0.05)	−0.03	−0.57 (−0.87 to −0.27)^a^	−0.12
Digit Span−Backward	0.51	3.30 (2.43 to 5.34)	0.55 (0.48 to 0.61)	0.56	−0.02 (−0.04 to −0.01)^a^	−0.07	0.08 (0.05 to 0.11)^a^	0.19	0.04 (−0.20 to 0.25)	0.01	−0.40 (−0.59 to −0.04)^a^	−0.10
Digit Span−Total	0.68	5.22 (2.95 to 7.75)	0.73 (0.68 to 0.78)	0.71	−0.02 (−0.05 to −0.01)^a^	−0.05	0.11 (0.06 to 0.16)^a^	0.11	−0.17 (−0.53 to 0.18)	−0.02	−0.67 (−1.12 to −0.21)^a^	−0.08
Trails A	0.54	1.88 (−6.41 to 9.38)	0.57 (0.47 to 0.68)	0.57	0.24 (0.15 to 0.34)^a^	0.14	−0.55 (−0.80 to −0.29)^a^	−0.16	0.30 (−1.21 to 1.72)	0.01	3.26 (1.60 to 4.95)^a^	0.11
Trails B	0.62	7.30 (−23.04 to 37.99)	0.58 (0.49 to 0.67)	0.58	1.05 (0.69 to 1.44)^a^	0.14	−3.26 (−4.40 to −2.16)^a^	−0.22	−1.02 (−6.82 to 4.74)	−0.01	6.97 (0.42 to 14.23)^a^	0.06
FAS	0.69	6.72 (1.15 to 12.02)	0.76 (0.71 to 0.81)	0.74	−0.02 (−0.07 to 0.81)	−0.01	0.36 (0.23 to 0.50)^a^	0.14	−0.25 (−1.22 to 0.66)	−0.01	−0.37 (−1.48 to 0.96)	−0.02
Animals	0.37	16.01 (10.94 to 19.77)	0.36 (0.25 to 0.54)	0.43	−0.11 (−0.15 to −0.07)^a^	−0.19	0.27 (0.16 to 0.35)^a^	0.25	−0.02 (−0.55 to 0.55)	0	−0.05 (−0.76 to 0.63)	−0.01
Digit Symbol Substitution	0.83	12.97 (7.41 to 18.39)	0.87 (0.82 to 0.92)	0.84	−0.13 (−0.18 to −0.07)^a^	−0.09	0.15 (0.03 to 0.28)^a^	0.05	0.75 (0 to 1.51)^a^	0.03	−1.03 (−2.04 to 0)^a^	−0.04

Abbreviations: FAS, phonemic fluency; MMSE, Mini Mental Status Exam; SEVLT, Spanish English Verbal Learning Test.

^a^ *P* < .05 for that predictor in the regression model.

When these SRB models were applied back to the intact sample, *z* scores were generated for each individual for the 13 cognitive scores. Comparisons between the non-Hispanic White and Hispanic individuals on those 13 SRB *Z* scores via independent *t* tests yielded no statistically significant differences (Table 4). When individual z scores were categorized (ie, decline/stable/improve) and the non-Hispanic White and Hispanic individuals were compared via χ^2^ analyses, the groups differed on 3 of the 13 cognitive change scores (MMSE, TMTA, TMTB). In each case, fewer-than-expected non-Hispanic White individuals showed improvement and more-than-expected Hispanic individuals showed decline (Table 4).

**Table 4.** Group Comparisons on SRB *z* Scores

Group	*z* Score Hispanic, mean (SD)	*z* Score non-Hispanic White, mean (SD)	*P* value	Categorized Hispanic, No.			Categorized Non-Hispanic White, No.			*P* value
				Decline	Stable	Improve	Decline	Stable	Improve	
MMSE Total	−0.01 (1.20)	−0.01 (0.80)	>.99	8^a^	88	5	3	97	0	<.001
SEVLT Learning	0 (1.04)	0.00 (0.96)	>.99	5	89	5	5	92	3	.14
SEVLT Delay	−0.01 (1.02)	−0.01 (0.98)	.99	6	91	4	5	92	3	.80
Logical Memory Immediate	0 (0.95)	0 (1.04)	>.99	3	94	3	6	90	4	.12
Logical Memory Delay	0 (0.98)	0 (1.01)	>.99	3	91	6	4	91	5	.75
Digit Span-Forward	0 (0.86)	0 (1.09)	.99	3	94	3	6	87	7	.01
Digit Span-Backward	−0.01 (0.88)	−0.01 (1.08)	.98	3	93	4	5	89	6	.19
Digit Span - Total	0.01 (0.83)	0.01 (1.11)	.98	3	95	2	5	88	7	.004
Trails A	0 (1.23)	0 (0.77)	>.99	10^a^	87	4	3	97	1	<.001
Trails B	0 (1.27)	0 (0.72)	>.99	9^a^	85	6	3	97	0	<.001
FAS	0 (0.91)	0 (1.06)	>.99	3	95	3	5	89	5	.03
Animals	0 (0.89)	0 (1.08)	.99	2	95	3	6	88	6	.007
Digit Symbol Substitution	−0.01 (1.28)	−0.01 (0.78)	.99	5	91	4	5	92	3	.68

Abbreviations: FAS, phonemic fluency; MMSE, Mini Mental Status Exam; SEVLT, Spanish English Verbal Learning Test.

^a^ *P* < .001 for comparisons between ethnic groups on the same column.

### Secondary Analyses

Generating the SRB models using only the non-Hispanic White individuals and applying them back to all individuals, the non-Hispanic White and Hispanic individuals differed on 9 of the 13 SRB *z* scores, with the non-Hispanic White showing significantly better change than the Hispanic individuals (Table 5). When the groups were compared on the categorized *z *scores, they significantly differed on 5 of the 13 cognitive change scores, with Hispanic participants showing more-than-expected decline compared with non-Hispanic White individuals (Table 5).

**Table 5.** Group Comparisons on SRB *z* Scores Developed on Only the Non-Hispanic White Sample

Group	*z* Score Hispanic, mean (SD)	*z* Score Non-Hispanic White, mean (SD)	*P *value^a^	Categorized Hispanic			Categorized Non-Hispanic White			*P *value^a^
				Decline	Stable	Improve	Decline	Stable	Improve	
MMSE Total	−.90 (1.65)	−0.04 (1.00)	<.001	24	76	0	6	94	0	<.001
SEVLT Learning	−0.42 (1.12)	−0.02 (1.00)	<.001	13	85	2	5	91	4	<.001
SEVLT Delay	−0.34 (1.07)	0.01 (1.00)	<.001	9	88	3	5	91	3	.10
Logical Memory Immediate	−0.34 (0.91)	0 (1.00)	<.001	8	91	1	6	91	3	.13
Logical Memory Delay	−0.28 (0.98)	0 (1.00)	<.001	7	90	3	4	92	5	.14
Digit Span-Forward	−0.21 (0.80)	0 (1.00)	<.001	3	95	1	5	90	5	.01
Digit Span-Backward	−0.08 (0.83)	−0.01 (1.00)	.28	4	94	3	4	92	4	.52
Digit Span-Total	0.03 (0.79)	0.01 (1.00)	.80	2	96	2	3	91	6	.01
Trails A	−0.90 (1.73)	0 (1.00)	<.001	25	72	3	6	92	3	<.001
Trails B	−0.67 (1.87)	0(1.00)	<.001	23	72	5	6	93	2	<.001
FAS	−0.08 (0.86)	0 (1.00)	.22	2	96	2	4	91	5	.04
Animals	−0.12 (0.87)	0 (1.00)	.08	3	95	2	4	91	5	.04
Digit Symbol Substitution	−0.47 (1.16)	0 (1.00)	<.001	13	85	2	5	90	5	<.001

Abbreviations: FAS, phonemic fluency; MMSE, Mini Mental Status Exam; SEVLT, Spanish English Verbal Learning Test.

^a^ *P *value for comparisons between ethnic and racial groups on SRB *z *scores and percentage decline, stable, and improve, respectively.

## Discussion

This article sought to establish SRB formulae for identifying clinically meaningful cognitive change in a community-based cohort of cognitively intact Hispanic adults and elders for several primary cognitive domains. Consistent with the hypothesis and cross-sectional studies on race and ethnicity, ethnicity was significantly associated with cognitive change over time for most of the measures. Across 10 of the 13 cognitive measures, ethnicity significantly contributed to predicted follow-up scores. In these models, Hispanic older adults were predicted to have poorer cognitive scores on follow-up than their non-Hispanic White counterparts. However, when these SRB models were applied back to the entire sample, the predicted follow-up scores matched the observed follow-up scores for both Hispanic and non-Hispanic White participants. Such findings highlight the importance of considering ethnicity and race when ascertaining change in cognitive test performance over time and including these factors in the development and validation of cognitive change formulae. Such steps can improve clinical care by bolstering the information used in clinical decision making and broadening research opportunities for this traditionally underrepresented but growing segment of the population.

As race and ethnicity each influence risk of developing dementia^11^ and rates of cognitive decline,^12,13^ this study adds to the limited literature on incorporating such demographic variables into the prediction of cognitive change. For example, RCIs have been created for Spanish-speaking cognitively intact adults tested twice across 1 to 2 weeks with the Spanish-language NIH Toolbox Cognition Battery.^19^ Additionally, SRBs were developed on 665 community-dwelling Hispanic adults followed in a longitudinal research study of cognition with a brief cognitive battery across approximately 7 years.^20^ However, the current study has advantages over these prior works, including clinically relevant follow-up period (eg, 24 months vs 1 to 2 weeks and 7 years in the prior studies) and a comprehensive cognitive battery enriched for sensitivity to neurodegenerative disease. Importantly, the current study also included both Hispanic and non-Hispanic White participants, whereas the prior studies included only Spanish-speaking individuals. As such, these prior studies were not able to examine the influence of race and ethnicity on change scores. Although the mechanism of action of race and ethnicity on cognitive change might remain unclear, these demographic variables may be proxies for other determinants of health, such as quality of education, neighborhood disadvantage, acculturation, perceived control, discrimination, and systemic racism, and bias in test construction.^15^ Future studies that incorporate samples with varying race and ethnicity are encouraged to consider these variables in the development of change scores.

To internally-validate these findings, the SRB models were also generated on the cognitively intact non-Hispanic White individuals, and then they were applied back to all participants. Consistent with the primary findings, the non-Hispanic White and Hispanic individuals differed on 9 of the 13 SRB *z* scores. In these comparisons, non-Hispanic White *z *scores were significantly higher or better than those of Hispanic individuals. These secondary analyses reflect most studies in the field, where change scores are developed in only 1 ethnic group^19,20^ and highlight the consequences of failing to consider race and ethnicity in the prediction of cognitive change. That is, developing formulae on only 1 racial or ethnic group but applying them to another group may bias the group on which the formulae were not developed. Again, such findings argue for the inclusion of multiple racial and ethnic groups in the assessment of cognitive change so that more accurate results can be observed.

Although these findings demonstrate the value and need to include race and ethnicity as predictors cognitive change, their influence was comparable or less than that of other demographic predictors. In the primary analyses, the standardized β coefficients for age averaged 11% of the variance of the follow-up scores, the standardized β coefficients for education averaged 16% of the variance, and the standardized β coefficients for sex averaged 4% of the variance (Table 3). The standardized β coefficients for race and ethnicity averaged 7% of the variance. Therefore, changes in follow-up scores due to the inclusion of race and ethnicity are expected to be relatively small. Similarly, all demographic variables add relatively small amounts of variance compared with the baseline (T1) scores, which accounted for approximately 60% of the variance of the T2 score. These findings are consistent with other studies developing SRB models.^21,22^

### Limitations

This study has limitations. First, these SRBs are most applicable to individuals who have similar demographic and evaluation characteristics as the current sample. As such, change scores are less applicable to those younger than 50 years of age, those with less than high school education, those who are neither non-Hispanic White nor Hispanic, and those with retest intervals much shorter or much longer than 2 years. Second, since these prediction formulae were developed on cognitively intact individuals, they would need to be validated in cognitively impaired individuals (eg, MCI or dementia) to demonstrate their clinical and research utility. Third, as displayed in Table 3, the SRBs do not capture all of the variance in predicting T2 scores (eg, 37% to 83% of variance). Therefore, future analyses should seek additional variables that might better predict follow-up scores (eg, sensory and physical functioning, medical comorbidities, quality of education, engagement and effort). Fourth, the normative data^18^ used to classify individuals as CU did not correct for race or ethnicity. As such, there may be a higher proportion of non-Hispanic White participants who are CU, but all participants in the CU category will fall within a similar absolute raw score range for age, education, and language of test administration. The subsequent group comparisons, including comparisons of SRBs, could be influenced by any bias in this normative data. Fifth, the language of test administration was not known for all individuals in the sample. This variable may also influence cognitive baseline and change scores, as much or more so than race or ethnicity. Finally, for the Hispanic participants (which is an ethnic group), information on their race was not collected. For the non-Hispanic White participants (which is a racial group), information on their ethnicity was not collected. As such, our analyses conflate ethnicity and race. Future studies should consider both language and more detailed information about race and ethnicity. Despite these limitations, the current results highlight the need to include race or ethnicity as a predictor of cognitive change, as well as the consequences of not doing so.

## Conclusions

Evaluating change over time in cognition is a common component of clinical practice and research, and differentiating significant and meaningful change from normal variability in test performance can be facilitated using empirically derived prediction models. SRB models can be developed to predict future performance based on prior performance and demographics, and current findings highlight the importance of including race and ethnicity in such models. This ability to accurately predict cognitive change will become increasingly important as clinical practice and clinical trials become more diverse and culturally appropriate in this burgeoning global medical and societal crisis.

## Notes

Supplement 1.
HABS-HD Group Members

Supplement 2.
Data Sharing Statement
