# Supplementary material for: Derivation of Indices of Cognitive Change Among Hispanic Adults and Elders
Source: JAMA Netw Open. 2024 Sep 3;7(9):e2431180. doi: 10.1001/jamanetworkopen.2024.31180 (PMC11372505; doi:10.1001/jamanetworkopen.2024.31180)
Supplement: Supplement 1. — HABS-HD Group Members [file jamanetwopen-e2431180-s001.pdf]

Supplemental Online Content: Nonauthor Collaborators
*First name, last name, and suffix (if applicable) are required and will appear in PubMed.

*Group Name(s): Health and Aging Brain Study-Health Disparities

***First Name and Middle Initial(s)**	***Last Name**	***Suffix (eg, Jr, III)**	Academic Degrees	Institution	Location (city, state/province, country)	Role or Contribution, eg, chair, principal investigator	Group (if more than 1 Group listed in the byline) and/or Subgroup (eg, Steering Committee)
Sid	O'Bryant		PhD	University of North Texas Health Science Center			
Kristine	Yaffe		MD	University of California - San Francisco			
Arthur	Toga		PhD	University of Southern California			
Robert	Rissman		PhD	University of Southern California			
Leigh	Johnson		PHD	University of North Texas Health Science Center			
Meredith	Braskie		PhD	University of Southern California			
Kevin	King		MD	Barrow Neurological Institute			
James R.	Hall		PhD	University of North Texas Health Science Center			
Melissa	Petersen		PhD	University of North Texas Health Science Center			
Raymond	Palmer		PhD	University of Texas Health Science Center - San Antonio			
Robert	Barber		PhD	University of North Texas Health Science Center			
Yonggang	Shi		PhD	University of Southern California			
Fan	Zhang		PhD	University of North Texas Health Science Center			
Rajesh	Nandy		PhD	University of North Texas Health Science Center			
Roderick	McColl		PhD	UT Southwestern Medical Center			
David	Mason		DO	University of North Texas Health Science Center			
Bradley	Christian		PhD	University of Wisconsin-Madison			
Nicole	Phillips		PhD	University of North Texas Health Science Center			
Stephanie	Large		PhD	University of North Texas Health Science Center			
Joe	Lee		DrPH	Columbia University			
Badri	Vardarjaran		PhD	Columbia University			
Monica	Rivera Mindt		PhD	Fordham University			
Amrita	Cheema		PhD	Georgetown University			
Lisa	Barnes		PhD	Rush University			
Mark	Mapstone		PhD	University of California - Irvine			
Annie	Cohen		PhD	University of Pittsburgh			
Amy	Kind		PhD	University of Wisconsin-Madison			
Ozioma	Okonkwo		PhD	University of Wisconsin-Madison			
Raul	Vintimilla		MD, MPH	University of North Texas Health Science Center			

Supplemental Online Content: Nonauthor Collaborators
*First name, last name, and suffix (if applicable) are required and will appear in PubMed.

***First Name and Middle Initial(s)**	***Last Name**	***Suffix (eg, Jr, III)**	Academic Degrees	Institution	Location (city, state/province, country)	Role or Contribution, eg, chair, principal investigator	Group (if more than 1 Group listed in the byline) and/or Subgroup (eg, Steering Committee)
Zhengyang	Zhou		PhD	University of North Texas Health Science Center			
Michael	Donohue		PhD	University of Southern California			
Rema	Raman		PhD	University of Southern California			
Matthew	Borzage		PhD	University of Southern California			
Michelle	Mielke		PhD	Wake Forest University			
Beau	Ances		PhD, MD	Washington University in Saint Louis			
Ganesh	Babulal		PhD	Washington University School of Medicine in Saint Louis			
Jorge	Llibre-Guerra		MD	Washington University School of Medicine in Saint Louis			
Carl	Hill		PhD, MPH	Alzheimer's Association			
Rocky	Vig		BS	Midtown Medical Imaging			
