# Supplementary material for: Derivation of Indices of Cognitive Change Among Hispanic Adults and Elders
Source: JAMA Netw Open. 2024 Sep 3;7(9):e2431180. doi: 10.1001/jamanetworkopen.2024.31180 (PMC11372505; doi:10.1001/jamanetworkopen.2024.31180)
Supplement: Supplement 2. — Data Sharing Statement [file jamanetwopen-e2431180-s002.pdf]

# Data Sharing Statement

Duff. Derivation of Indices of Cognitive Change Among Hispanic Adults and Elders. *JAMA Netw Open*. Published September 03, 2024. doi:10.1001/jamanetworkopen.2024.31180

**Data**
**Data available:** Yes
**Data types:** Deidentified participant data
**How to access data:** https://apps.unthsc.edu/itr/data-list
**When available:** With publication

**Supporting Documents**
**Document types:** None

**Additional Information**
**Who can access the data:** researchers whose proposed use of the data has been approved
**Types of analyses:** for a specified purpose
**Mechanisms of data availability:** after approval of a proposal, or with a signed data access agreement
